# Assessing what matters most in older emergency department patients

**DOI:** 10.1093/ageing/afaf334

**Published:** 2025-11-18

**Authors:** Johannes Aemilius Griese, Luca Ünlü, James van Oppen, Noemi Bühler, Noah Maximilian Henz, Thomas Dreher-Hummel, Florian Grossmann, Georgiana Virant, Roland Bingisser, Christian Hans Nickel

**Affiliations:** Department of Emergency Medicine, University Hospital Basel, University of Basel, Petersgraben 2, Basel, Basel-Stadt 4031, Switzerland; Department of Emergency Medicine, University Hospital Basel, University of Basel, Petersgraben 2, Basel, Basel-Stadt 4031, Switzerland; Centre for Urgent and Emergency Care Research, University of Sheffield, S1 4DA, Sheffield, England, UK; Department of Emergency Medicine, University Hospital Basel, University of Basel, Petersgraben 2, Basel, Basel-Stadt 4031, Switzerland; Department of Emergency Medicine, University Hospital Basel, University of Basel, Petersgraben 2, Basel, Basel-Stadt 4031, Switzerland; Department of Emergency Medicine, University Hospital Basel, University of Basel, Petersgraben 2, Basel, Basel-Stadt 4031, Switzerland; Department of Acute Medicine, University Hospital Basel, Basel, Basel-Stadt, Switzerland; Department of Emergency Medicine, University Hospital Basel, University of Basel, Petersgraben 2, Basel, Basel-Stadt 4031, Switzerland; Department of Emergency Medicine, University Hospital Basel, University of Basel, Petersgraben 2, Basel, Basel-Stadt 4031, Switzerland; Department of Emergency Medicine, University Hospital Basel, University of Basel, Petersgraben 2, Basel, Basel-Stadt 4031, Switzerland

**Keywords:** emergency department, older adults, patient-centred care, what matters most, frailty

## Abstract

**Background:**

Older emergency patients have complex health needs and diverse personal priorities not captured by traditional single-disease approaches. Asking ‘what matters most’ may facilitate a more patient-centred approach. However, conceptual frameworks to document patient values have neither been implemented nor operationalised for use in the emergency department (ED).

**Objective:**

To investigate the feasibility of asking ‘what matters most’ in the ED, assess patient priorities and determine the utility of a conceptual framework for documenting these.

**Methods:**

Prospective, observational study in a Swiss ED with consecutive patients aged ≥65 years. Feasibility was determined as proportion of included patients to eligible patients. Patient responses were categorised using a conceptual framework consisting of 8 domains: *principles, relationships, emotions, activities, abilities, possessions, medical and others*. Framework evaluation included interrater reliability (IRR), time-to-abstraction rate and a questionnaire assessing utility of the framework.

**Results:**

Asking what ‘matters most’ was feasible, including 1349 of 1625 patients (83.0%). Regarding categories of the conceptual framework, 504 patients (37.4%) reported medical issues, 297 (22.0%) relationships, 268 (19.9%) abilities and 154 (11.4%) emotions as their priority. Patients aged ≥85 years or having frailty more frequently prioritised abilities and emotions, whereas patients 65–84 years or without frailty prioritised medical issues. The framework showed substantial IRR (κ = 0.668), good time-to-abstraction rates and high ratings in the utility questionnaire.

**Conclusions:**

Asking older people ‘what matters most’ is feasible and potentially useful in the ED setting. Applying a conceptual framework enables systematic documentation and may support patient-centred and holistic emergency care.

## Key Points

- Asking patients ‘What matters most to you?’ is feasible and potentially useful in the emergency department (ED) setting.
- Many older patients prioritise relationships, functional abilities and emotional well-being over medical concerns.
- Asking ‘what matters most’ in the ED may help tailor treatment decisions and service design towards patient-centredness.
- A conceptual framework enables systematic documentation and may support patient-centred and holistic care.
- Patient priorities change between ED visits, emphasising the need for repeated value elicitation.

## Introduction

The number of older people visiting an emergency department (ED) is increasing [1]. In Europe, 40% of older adults presenting to the ED live with at least mild frailty [2]. The single-disease approaches commonly applied in the ED fail to address the complexities in patients living with frailty. In older people with frailty, a more patient-centred, holistic approach to care is required [3–9].

However, the majority of available approaches lack pragmatism and feasibility given the limited resources and time constraints in the ED [3, 4]. In particular, the multi-dimensional, multi-professional and longitudinal comprehensive geriatric assessment (CGA) might be difficult to deliver in the fast-paced and often crowded ED setting [3, 4, 10]. The ‘5 M Framework’—mobility, mind, medication, multimorbidity and what matters most—offers a structured yet adaptable entry point whilst remaining feasible and useful for the ED setting. In this way, the core principles of CGA can be incorporated through pragmatic interventions, without the need for the longitudinal infrastructure of a traditional CGA [3–5, 11]. In this context, the question on ‘what matters most’ might help instil a more patient-centred mindset [12]. Despite the establishment of patient-centred care as a ‘vital aim’ of quality health care [13, 14], the assessment and documentation of what ‘matters most’ remains underrepresented in clinical practice [15]. Rethinking service designs by prioritising outcomes that matter most to patients may improve both effectiveness of healthcare services and systems as well as patient experience, e.g. by preventing potentially burdensome interventions and potentially avoidable hospital admissions, whilst ensuring access to effective treatment aligned with patients’ needs and healthcare goals [6–8, 16–18]. Whilst ‘matters most’ elicitation serves as a pragmatic entry point to a holistic assessment, conceptual frameworks might further support practitioners in reflecting upon outcome goals with patients and thereby facilitating a structured documentation [3]. For this purpose, previous studies have identified personal values through qualitative interviews with older people living with frailty conducted in various acute settings [12, 19–21]. However, such conceptual frameworks have not been implemented or operationalised for clinical or research purposes in the ED setting to date.

One such conceptual framework by Lim et al. originally consists of 6 domains: ‘principles’ (core values that guide life), ‘relationships’ (connections with others like family and friends), ‘emotions’ (personal feelings and moods), ‘activities’ (things which are done for work or leisure), ‘abilities’ (mental or physical skills) and ‘possessions’ (valued items or spaces). This conceptual framework is based on qualitative interviews with patients living with multiple chronic conditions [21]. Its clear structure and relevant themes suggest potential for use in the ED setting as an adjunct to early elicitation of patient values and outcome goals.

The objective of this study was to investigate the potential use of a conceptual framework to document meaningful patient values in the emergency setting, in order ultimately to aid delivery of goal-based comprehensive care. The aims were, first, to test the feasibility of asking ‘What matters most to you?’ of a large consecutive sample of patients aged 65 years and older presenting to the ED. Secondly, we aimed to categorise these personal values using Lim’s conceptual framework [21], and to examine their distribution for potential associations with patient characteristics. Thirdly, we aimed to evaluate the conceptual framework for feasibility of clinical use through interrater reliability (IRR), time-to-abstraction and questionnaire assessment of usability, testability, applicability and familiarity in an exploratory fashion. Lastly, we investigate whether patient priorities changed in those presenting to the ED more than once.

## Methods

### Study design and setting

This prospective, observational study included consecutive patients aged 65 and older presenting to the ED of a single academic tertiary care hospital in Switzerland, between 15 April and 27 May 2024. Patient recruitment was continuous, 24 hours a day, seven days a week, throughout the study period. The University Hospital Basel ED treats >56 000 patients per year, of whom 18 000 are ≥65 years of age. Of those, about a third are living with frailty [22]. Patients with ophthalmological emergencies are managed in separate facility outside of the ED and were therefore excluded from this study.

The study protocol was approved by the local ethics committee (EKNZ No. 236/13). The study is registered at ClinicalTrials.gov (NCT05400707) and was conducted in accordance with the Declaration of Helsinki.

### Selection of participants

During the study period, all patients aged ≥65 years presenting to the ED of the University Hospital Basel were potentially eligible for inclusion. Exclusion criteria comprised inability or unwillingness to provide informed consent verbally or inability to communicate with study staff (e.g. due to treatment in the resuscitation area, altered mental status or language barriers). Additionally, patients assigned Emergency Severity Index (ESI) levels 1 (life-saving interventions required) or 5 (lowest urgency, see-and-treat pathway with very short ED stays) were excluded to avoid disruption of ED processes.

### Data collection

Each participant was asked two open-ended questions: ‘What matters most to you at the moment?’ and ‘Why is that important to you?’ [12]. All study data were prospectively collected using case report forms (CRFs) specifically designed for this study and integrated into the hospital information system (Ismed®, ProtecData, Boswil, Switzerland). Study staff recording the data received structured training consisting of two dedicated sessions led by the principal investigator, a senior physician, covering key geriatric concepts as well as training on communication with older patients. Competence and adherence to the framework were monitored by the study team during regular shift briefings, enabling continuous feedback and support. Patient responses were documented in a narrative fashion in a free-text field without word limits. Demographic information, including age, sex and ESI triage levels, ranging from 1 (highest urgency) to 5 (lowest urgency), were automatically extracted from the electronic health record (EHR) into the CRF. The clinical frailty scale (CFS) was assigned by triage clinicians, and frailty was considered as CFS ≥5 [23, 24]. Study staff were present 24/7 during the study period to conduct patient interviews.

To categorise patient responses, we employed the conceptual framework developed by Lim et al. [21], which was selected a priori. The Lim framework was originally designed to better understand what patients with complex health needs consider most important in their daily lives and to support value-concordant care planning. This conceptual framework identifies six domains of personal values in patients with multimorbidity: *Principles*, *Relationships*, *Emotions*, *Activities*, *Abilities* and *Possessions*. Two independent reviewers (NB, NH) categorised all responses according to this conceptual framework. Discrepancies were resolved by a third reviewer (GV), who provided the final categorisation. All three reviewers were master’s-level medical students who underwent structured training in the application of the conceptual framework. The abstraction process was conducted using Research Electronic Data Capture, hosted by the Department of Clinical Research at the University of Basel. All reviewers were blinded to each other’s assessments.

Since all patient responses were collected in German, the Lim et al. framework was translated into German following International Society for Pharmacoeconomics and Outcomes Research guidelines, using a forward- and backward translation process [25]. In total, 11 of the recommended 12 chart review criteria were adhered to (for reference see [26, 27]). Due to the nature of the study and abstraction task, it was not possible to blind the abstractors to the study hypothesis. Training of chart abstractors was conducted using a sample of practice cases from patients who were not part of the study population. Based on these, we observed that some patients prioritised medical issues. Hence, an additional category, ‘medical’, was introduced to reflect the specific relevance of medical concerns in the ED context, acknowledging the considerations of reversibility in this setting compared to palliative care. We also added the variable ‘other’ to accommodate responses that did not fit the predefined conceptual framework, ensuring that patients’ input was accurately abstracted in case it did not align with one of the domains.

After completing the abstraction process, a questionnaire was distributed to all three abstractors. The questionnaire comprised four questions for each of the four evaluation criteria: usability, testability, applicability and familiarity (see Appendix Table 1). Each question was rated on a four-point rating scale scored 1 (strongly disagree) to 4 (strongly agree).

### Outcomes

Feasibility was assessed by the proportion of patients whose responses were included in the study out of those who were eligible. Secondary outcomes were the quantification of abstracted responses by using the adapted conceptual framework and the impact of age, sex, ESI triage level and frailty on patient priorities. The adapted conceptual framework was assessed through IRR, the time-to-abstraction rate and a questionnaire evaluating abstractors’ perceptions. We selected IRR as it reflects the extent to which a tool is understandable and practicable. A high IRR indicates that the framework is consistently understood and applied by all abstractors, thereby supporting its practical usability. In addition, the time-to-abstraction rate, defined as the number of abstractions completed per minute, was used to estimate efficiency. For the questionnaire, we selected domains that reflect criteria proposed by the Theory Comparison and Selection Tool, which offers a structured approach for evaluating and systematically assessing frameworks [28]. These are: Usability (clarity, structure and ease of use, including how intuitively statements could be categorized), testability (clarity and consistency of categorisation criteria, as well as justifiability and reproducibility), applicability (practical relevance and flexibility of the framework in capturing real-world patient statements) and familiarity (how quickly and easily users can understand and apply the framework). Finally, we assessed whether patient priorities changed amongst those with ED revisits during the 30-day study period.

### Statistical analysis

Statistical analysis was conducted using R studio software (version 4.4.1) [29]. For the primary outcome, we calculated the proportion of patients who were included in the study in relation to those who were eligible, expressed as counts and percentages. For the secondary outcome, descriptive statistics were used to summarise the study population and reported outcomes. Categorical variables are presented as total counts and percentages. Continuous variables are presented as medians with interquartile ranges (IQR), as appropriate. No inferential statistical tests were performed, as the analysis was descriptive in nature. Baseline demographics were analysed only for each patient’s first presentation. Subsequent presentations within the study period were excluded from the feasibility analysis but were analysed separately to explore potential changes in patients’ priorities based on their responses to ‘what matters most’. To assess the IRR of the categorisation process, an unweighted Cohen’s Kappa was calculated between the two reviewers who categorized all responses. A Cohen’s kappa value <0.00 was considered poor agreement, 0.00–0.20 slight agreement, 0.21–0.40 fair agreement, 0.41–0.60 moderate agreement, 0.61–0.80 substantial agreement and 0.81–1.00 almost perfect agreement [30, 31]. Time-to-abstraction rate was defined as the number of patient responses that were abstracted into the framework per minute, serving as a measure of coding efficiency during the framework application. Responses to the questionnaire on usability, testability, applicability and familiarity were summarised descriptively and presented as mean scores (range, 1–4) for the single topics. Each item was rated on a four-point rating scale: strongly disagree (1), somewhat disagree (2), somewhat agree (3) and strongly agree (4). A four-point rating scale was chosen to prevent neutral responses. This evaluation was exploratory in nature, intended to provide preliminary insights into efficiency, usability, testability, applicability and familiarity.

## Results

**Figure 1:**
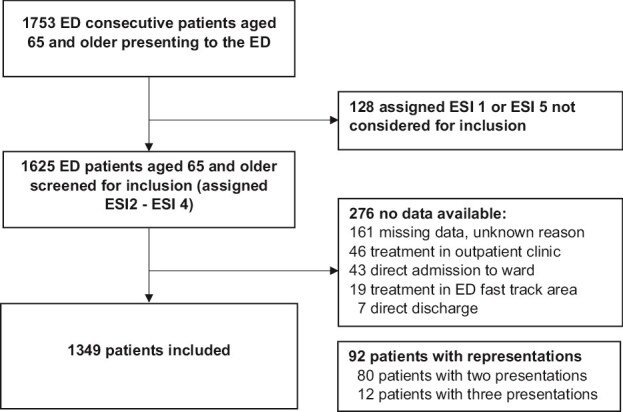
Flowchart showing inclusion of 1349 patients and reasons for exclusion; 92 patients had repeat presentations.

During the study period, 1753 patients aged 65 and older presented to the ED of whom 1625 patients met the inclusion criteria. Data were unavailable for 276 patients; detailed reasons for exclusion are provided in Figure 1. The final analysis included 1349 patients, of whom 419 (31.1%) were living with frailty (CFS ≥ 5). Median age of the study population was 78 years (IQR, 72; 84 years), and 54.0% (*n* = 728) were female (see Table 1).

**Table 1:** Baseline characteristics of the study population.

	All patients (*N* = 1349)
Age, median (IQR)	78.00 (72.00; 84.00)
Sex (F), n (%)	728 (54.0)
Emergency severity index (ESI)	
ESI 2, n (%)	583 (43.2)
ESI 3, n (%)	682 (50.6)
ESI 4, n (%)	84 (6.2)
CFS	
CFS 1 very fit, n (%)	55 (6.4)
CFS 2 Well, n (%)	219 (15.8)
CFS 3 managing well, n (%)	366 (26.5)
CFS 4 Vulnerable, n (%)	232 (16.8)
CFS 5 mildly frail, n (%)	201 (14.5)
CFS 6 moderately frail, n (%)	142 (10.3)
CFS 7 severely frail, n (%)	68 (4.9)
CFS 8 very severely frail, n (%)	5 (0.4)
CFS 9 terminally ill, n (%)	3 (0.2)
CFS missing data	58 (4.2)

### Feasibility

Of 1625 eligible patients, asking ‘what matters most’ was feasible in 1349 (83.0%) of patients.

### Assessing what ‘matters most’

In the study cohort, 504 (37.4%) of patients identified medical issues, such as receiving a diagnosis, accessing treatment or alleviating symptoms as their foremost concern. Relationships were prioritised by 297 (22.0%) of patients, whilst 268 (19.9%) emphasised the importance of maintaining their abilities. Additionally, 154 (11.4%) reported that emotions were their primary concern. Fewer patients identified activities, principles or possessions as their top priority. The various domains, together with their explanations and examples, are presented in Table 2. A detailed distribution of responses is presented in Table 3.

**Table 2:** Adapted framework based on Lim et al. including additional category ‘medical’, with definitions and examples.

Domain	Definition	Examples
Principles	Standards or values by which one should live, including aspirations (e.g. spirituality, independence, truth)	‘I want to have the feeling of being needed by society’, ‘that I can still say my evening prayer today and that the examination takes place beforehand, because I am religious, and I don’t want to miss evening prayer because I do it every evening’
Relationships	Connections to others (e.g. family, friends, community)	‘I want to go home, so I can look after my dog’; ‘I want to get home as quickly as possible, I am worried about my wife, living with a disability at home.’
Emotions	Feelings or moods; personal, embodied, or experiential states (e.g. success, convenience, calmness)	‘I want joy in life, I want to be happy’, ‘I need support with the overnight stay I feel unsafe’
Activities	Occupations, things people do for work or leisure (e.g. reading, gardening, self-care)	‘That I can go home and go for a walk again. Walking keeps me grounded.’; ‘I want to continue to work as an engineer, it gives my life meaning’
Abilities	Physical or mental abilities or skills (e.g. mental acuity, mobility, vision, problem-solving skills)	‘I would like to be able to walk safely, to maintain independence’; ‘No more dizziness, I feel unsafe. I enjoy driving car and motorbike, I want to feel safe on the road again’
Possessions	Material things that are kept, owned or valued, including premises and belongings (e.g. computer, 55 Chevy, home, Woodworking workshop woodshop)	‘Get home today if possible, so that the flat is not alone, otherwise the heating will be on’; ‘I want to go home and back to the garden, I have a large garden with fishes’
Medical	Getting a diagnosis, being treated, ease of symptoms	‘My back pain should go away, so that I am pain-free again’; ‘I don’t want to feel any pain, I want to be healthy’

**Table 3:** Quantification of `matters most` mapped to the adjusted Lim et al. framework

n (%)		Sex		Age			ESI score			Frailty^a^	
Category	All	Female	Male	65–74	75–84	≥85	ESI 2	ESI 3	ESI 4	CFS <5	CFS 5+
Medical	504 (37.4)	266 (36.5)	238 (38.3)	199 (41.0)	212 (38.8)	93 (29.3)	222 (38.1)	250 (36.7)	32 (38.1)	332 (38.1)	149 (35.6)
Relationships	297 (22.0)	160 (22.0)	137 (22.1)	103 (21.2)	119 (21.8)	75 (23.7)	142 (24.4)	142 (20.8)	13 (15.5)	200 (22.9)	84 (20.0)
Abilities	268 (19.9)	154 (21.2)	114 (18.4)	87 (17.9)	111 (20.3)	70 (22.1)	108 (18.5)	140 (20.5)	20 (23.8)	168 (19.3)	88 (21.0)
Emotions	154 (11.4)	83 (11.4)	71 (11.4)	58 (11.9)	51 (9.3)	45 (14.2)	58 (9.9)	82 (12.0)	14 (16.7)	87 (10.0)	60 (14.3)
Activities	74 (5.5)	31 (4.3)	43 (6.9)	28 (5.8)	29 (5.3)	17 (5.4)	32 (5.5)	39 (5.7)	3 (3.6)	54 (6.2)	17 (4.1)
Principles	24 (1.8)	15 (2.1)	9 (1.4)	7 (1.4)	9 (1.6)	8 (2.5)	12 (2.1)	11 (1.6)	1 (1.2)	15 (1.7)	9 (2.1)
Possessions	11 (0.8)	7 (1.0)	4 (0.6)	1 (0.2)	4 (0.7)	6 (1.9)	5 (0.9)	6 (0.9)	0 (0.0)	7 (0.8)	4 (1.0)
Other	17 (1.3)	12 (1.6)	5 (0.8)	3 (0.6)	11 (2.0)	3 (0.9)	4 (0.7)	12 (1.8)	1 (1.2)	9 (1.0)	8 (1.9)

The table shows total counts and percentages of categories by patient sex, age group, Emergency Severity Index (ESI) and Frailty according to the CFS.

^a^58 Missing Data for CFS scores

### Impact of age, sex, ESI triage level and frailty on ‘matters most’

To further explore factors influencing what ‘matters most’ to patients, we analysed whether age, sex, ESI triage level and frailty had an impact on patients’ priorities. Patients aged ≥85 years less frequently prioritised medical issues compared to those aged 65–74 and 75–84 years. Instead, they more often emphasised the importance of their abilities. Other categories showed only minor variations across age groups (see Appendix Figure 1).

Triage levels did not have an impact on the prioritisation of medical issues. However, patients with lower ESI scores (indicating higher urgency) more frequently emphasised relationships and less frequently prioritised abilities. In contrast, patients with higher ESI scores (indicating lower urgency) more frequently prioritised emotions (see Appendix Figure 2).

Patients who had frailty (CFS 5+) less frequently prioritised medical issues, relationships and activities compared to those without frailty. Instead, they more frequently emphasised the importance of their ‘abilities’ and ‘emotional’ aspects. Differences in the other categories were minor between the two groups (see Appendix Figure 3).

Patient sex exerted minimal influence on patient priorities in our cohort.

### Framework evaluation

The agreement between the abstractors was substantial with a Kappa coefficient of 0.668. Time-to-abstraction rates were calculated only for the two abstractors who reviewed all cases (N.B. and N.H.), yielding rates of 3.74 and 3.05 abstractions per minute, respectively, corresponding to less than 20 seconds per abstraction. Questionnaire results indicate an overall positive evaluation of the framework, with testability receiving the highest mean rating (3.75), followed by usability and familiarity (both 3.67), and applicability (3.58) (see Appendix Figure 4).

### Representations

**Figure 2:**
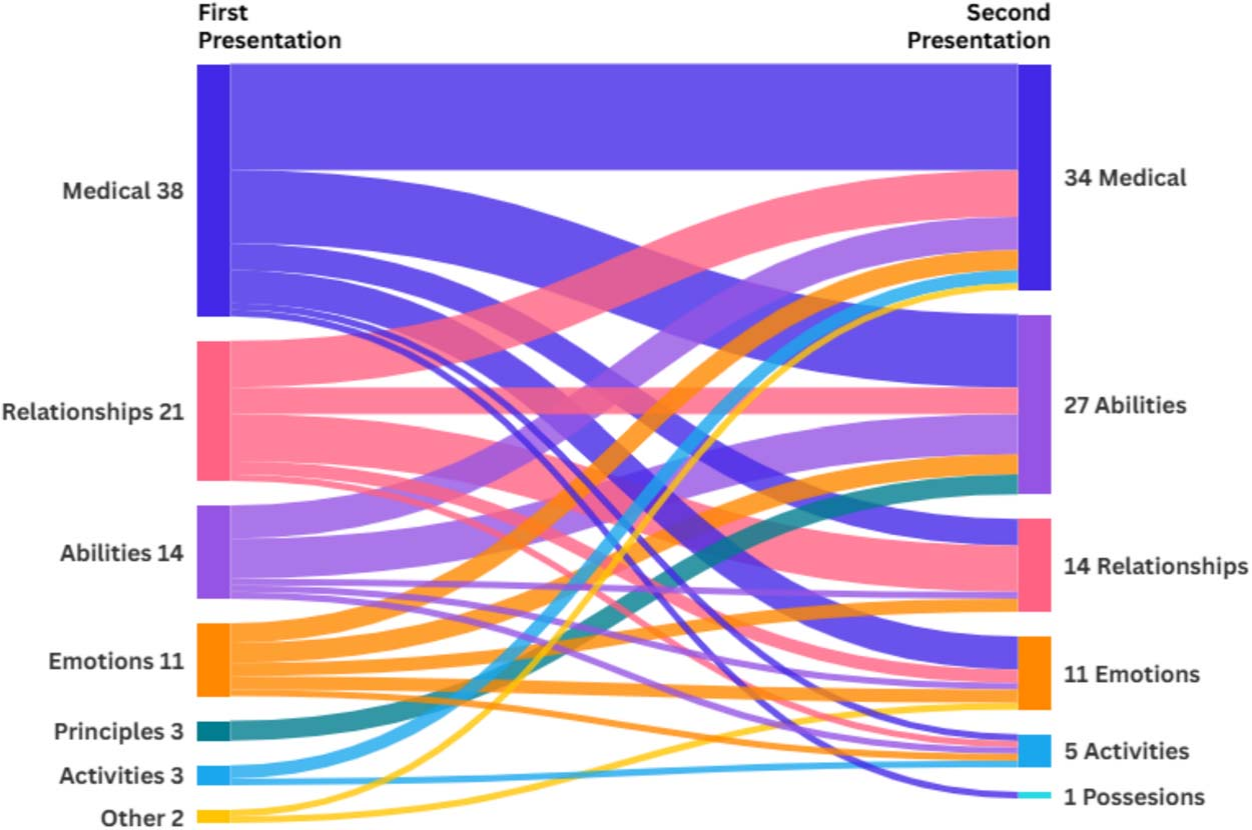
Sankey chart showing changes of ‘what matters most’ categories from the first to the second presentation. First and second presentations of representations (*N* = 92)

In our study population, 92 patients presented to our ED more than once. Amongst these, 60 (65.2%) reported different priorities during their second visit compared to their first (see Figure 2).

## Discussion

In this study we examined the feasibility of asking ‘what matters most?’, in a consecutive sample of patients aged ≥65 years in the ED setting. Our findings suggest not only that it is feasible to ask ‘what matters most?’ in the ED, but also that the adapted Lim framework might support consistent documentation of responses, thereby enabling systematic integration of service design adaptations.

Consistent with previous studies, we observed that not only asking ‘What matters most to you?’ but also following up with ‘Why does it matter most to you?’ further encourages patients to articulate their core motivations and meaningful outcomes [12]. This approach may offer a more pragmatic and feasible alternative to complex communication protocols.

Interestingly, only 37% of patients identified medical issues as their primary priority. Instead, many articulated preferences on relationships, functional ability and emotional well-being. Non-medical domains can be challenging to manage in emergencies. Where these are identified as mattering most, healthcare professionals will likely seek to follow with patient-based inquiry through clinical conversation, using the category as a starting point. These findings highlight the need to move beyond a single-disease approach in the ED and focus on the aspects that truly matter to older patients, underscoring the importance a more holistic, patient-centred approach to emergency care [3, 4, 7, 16].

Prioritisation of medical concerns decreased with age from 41% in the youngest category to 29% in the oldest. Additionally, 38% of non-frail versus 36% of frail people considered medical issues to matter most. Although the tendency towards deprioritisation of medical concerns was similar in both older and frail patients, the effect was more pronounced with increasing age than with frailty status. This finding is in line with previous work suggesting that goals of care might not be related to frailty status [32, 33]. But these are exploratory analyses and require further study.

Of note, in patients with repeat ED visits during the study period, 65.2% (60 out of 92) changed their stated priorities between presentations. This finding reinforces the idea that patient values are not fixed traits, but dynamic, context-dependent expressions which might be influenced by acute symptoms, situational stressors or an emotional state [34]. As such, it might be insufficient to capture patient preferences only once; instead, a repeat inquiry seems to be essential to align care with current priorities.

Collectively, these results support the integration of asking ‘What matters most to you?’ as a key to a more comprehensive assessment, enabling the delivery of more meaningful and individualised care. The pragmatic nature of this approach, along with its feasibility and proportionality to the emergency care context, combined with its ability to uncover patient priorities, position it as a potentially valuable tool in the ED [5]. We found that a majority of our patients were both willing and able to engage in this dialogue. The insights gained could potentially directly inform shared decision-making and care planning.

In summary, asking older ED patients ‘what matters most’ is a feasible and efficient intervention with the potential to enhance the quality of emergency care. Additionally, the use of a conceptual frameworks seems to support documentation and may help inform adjustments in service design.

Future research should focus on validating this framework and testing its generalisability, clinical utility, reliability and acceptability across diverse populations and healthcare systems in different countries. Furthermore, the actual impact of age and frailty status on patient priorities should be investigated further. Understanding patient outcome gaols is a core requisite of goal-based and person-centred care. That we have found that goals change over time reiterates the importance of eliciting these during every encounter. Aids to goal elicitation such as this framework have potential applications for further study such as patient-clinician communication support, service—level reinforcement of person-centred care processes, and system-level education and workforce development around the domains of care considered important by patients.

## Limitations

Despite its strengths, this study has several limitations. First, categorising patient responses within a predefined conceptual framework involved researcher interpretation. Although steps were taken to ensure consistency and transparency, some bias may remain, as the abstraction process could reflect researchers’ viewpoints alongside those of patients. Furthermore, the framework was originally developed for patients with multimorbidity and required adaptations for the emergency care setting. However, it appeared pragmatic and appropriate to the ED context [5].

Second, the question was posed by study staff rather than the treating team. Whilst our findings show that asking patients about ‘what matters most’ is feasible in the emergency setting, feasibility and acceptability may differ when implemented by clinical staff in routine care.

Third, this study was conducted in a tertiary care centre in Switzerland. Generalisability is limited due to the monocentric design. The predominantly Caucasian population, lower proportions of higher CFS values [35], and the specific organisational context may limit generalisability to other institutions, regions or more ethnically diverse healthcare systems.

Fourth, the exclusion of patients requiring life-saving interventions (ESI 1) or very well (ESI 5) patients, in order not to disrupt usual care, might lead to some selection bias. Patients assigned ESI 1 are typically in critical conditions (shock, trauma or cardiopulmonary resuscitation). Patients assigned ESI level 5 follow a see and treat pathway, not requiring external resources or hospitalisation. These patients receive minor assessments (blood pressure measurement or prescription of medications). Their consultation is usually closed at triage.

Fifth, only three abstractors were involved in the study, limiting the diversity of perspectives from which the abstraction process and framework applicability could be assessed. However, the small number allowed close monitoring, consistent training and in-depth feedback, which may have enhanced coherence and depth of evaluation.

## Supplementary Material

Supplementary_materials_afaf334

## Data Availability

The data are available from the corresponding author upon reasonable request.

## References

[1] Karamercan MA, Dündar DZ, Slagman A et al. Epidemiology of geriatric patients presenting to emergency departments in Europe: EGERS study. Eur J Emerg Med 2023;30:117–24. 10.1097/MEJ.0000000000000997. 36719188

[2] European taskforce on geriatric emergency medicine (ETGEM) collaborators. Prevalence of frailty in European emergency departments (FEED): An international flash mob study. Eur Geriatr Med 2024;15:463–70. 10.1007/s41999-023-00926-3. 38340282 PMC10997678

[3] Hogervorst VM, Buurman BM, De Jonghe A et al. Emergency department management of older people living with frailty: A guide for emergency practitioners. Emerg Med J EMJ 2021;38:724–9. 10.1136/emermed-2020-210014. 33883216

[4] Lucke JA, Mooijaart SP, Heeren P et al. Providing care for older adults in the emergency department: Expert clinical recommendations from the European task force on geriatric emergency medicine. Eur Geriatr Med 2022;13:309–17. 10.1007/s41999-021-00578-1. 34738224 PMC8568564

[5] Mooijaart SP, Lucke JA, Brabrand M et al. Geriatric emergency medicine: Time for a new approach on a European level. Eur J Emerg Med 2019;26:75–6. 10.1097/MEJ.0000000000000594. 30801429

[6] Van Oppen JD, Coats T, Conroy S et al. Person-centred decisions in emergency care for older people living with frailty: Principles and practice. Emerg Med J 2024;41:694–9. 10.1136/emermed-2024-213898. 39060102

[7] Tinetti ME, deCardi HM, Ejem D. One size fits all—An underappreciated health inequity. *JAMA*. Intern Med 2024;184:7. 10.1001/jamainternmed.2023.6035. 37983054

[8] Tinetti ME, Naik AD, Dindo L et al. Association of Patient Priorities—Aligned decision-making with patient outcomes and ambulatory health care burden among older adults with multiple chronic conditions: A nonrandomized clinical trial. JAMA Intern Med 2019;179:1688. 10.1001/jamainternmed.2019.4235. 31589281 PMC6784811

[9] Tinetti ME, Costello DM, Naik AD et al. Outcome goals and health care preferences of older adults with multiple chronic conditions. JAMA Netw Open 2021;4:e211271. 10.1001/jamanetworkopen.2021.1271. 33760091 PMC7991967

[10] European Task Force for Geriatric Emergency Medicine, European Society for Emergency Medicine, European Geriatric Medicine Society . Comprehensive geriatric assessment in the emergency department. [Poster] GeriEM Eur. Available from: https://posters.geriemeurope.eu/posters/p01/.

[11] Megalla M, Avula R, Manners C et al. Using the 4M model to screen geriatric patients in the emergency department. J Geriatr Emerg Med 2021;2:1013. 10.17294/2694-4715.1013.

[12] van den Ende ES, Schouten B, Kremers MNT et al. Understanding what matters most to patients in acute care in seven countries, using the flash mob study design. BMC Health Serv Res 2021;21:474. 10.1186/s12913-021-06459-4. 34011321 PMC8132421

[13] Shankar KN, Bhatia BK, Schuur JD. Toward patient-centered care: A systematic review of older adults’ views of quality emergency care. Ann Emerg Med 2014;63:529–550.e1. 10.1016/j.annemergmed.2013.07.509. 24051211

[14] Chassin MR . The urgent need to improve health care QualityInstitute of medicine national roundtable on health care quality. JAMA 1998;280:1000. 10.1001/jama.280.11.1000. 9749483

[15] Welch SA, Archer KR, Hymel AM et al. Hospital 4Ms: Documentation and association with patient characteristics. J Am Geriatr Soc 2025;73:172–81. 10.1111/jgs.19205. 39373341 PMC11734090

[16] Conroy SP, Van Oppen JD. Are we measuring what matters to older people? Lancet Healthy Longev 2023;4:e354–6. 10.1016/S2666-7568(23)00084-3. 37336229

[17] Tinetti ME, Hashmi A, Ng H et al. Patient priorities—Aligned care for older adults with multiple conditions: A nonrandomized controlled trial. JAMA Netw Open 2024;7:e2352666. 10.1001/jamanetworkopen.2023.52666. 38261319 PMC10807252

[18] Mitchell SL, Teno JM, Kiely DK et al. The clinical course of advanced dementia. N Engl J Med 2009;361:1529–38. 10.1056/NEJMoa0902234. 19828530 PMC2778850

[19] Gettel C, Venkatesh A, Dowd H et al. A qualitative study of “what matters” to older adults in the emergency department. West J Emerg Med 2022;23:579–88. 10.5811/westjem.2022.4.56115. 35980413 PMC9391017

[20] van Oppen JD, Coats TJ, Conroy SP et al. What matters most in acute care: An interview study with older people living with frailty. BMC Geriatr 2022;22:156. 10.1186/s12877-022-02798-x. 35216550 PMC8880299

[21] Lim CY, Berry ABL, Hirsch T et al. Understanding what is most important to individuals with multiple chronic conditions: A qualitative study of patients’ perspectives. J Gen Intern Med 2017;32:1278–84. 10.1007/s11606-017-4154-3. 28849368 PMC5698221

[22] Kaeppeli T, Rueegg M, Dreher-Hummel T et al. Validation of the clinical frailty scale for prediction of thirty-day mortality in the emergency department. Ann Emerg Med 2020;76:291–300. 10.1016/j.annemergmed.2020.03.028. 32336486

[23] Rockwood K, Song X, MacKnight C et al. A global clinical measure of fitness and frailty in elderly people. Can Med Assoc J 2005;173:489–95. 10.1503/cmaj.050051. 16129869 PMC1188185

[24] Pulok MH, Theou O, van der Valk AM et al. The role of illness acuity on the association between frailty and mortality in emergency department patients referred to internal medicine. Age Ageing 2020;49:1071–9. 10.1093/ageing/afaa089. 32392289 PMC7583513

[25] Wild D, Grove A, Martin M et al. Principles of good practice for the translation and cultural adaptation process for patient-reported outcomes (PRO) measures: Report of the ISPOR task force for translation and cultural adaptation. Value Health 2005;8:94–104. 10.1111/j.1524-4733.2005.04054.x. 15804318

[26] Worster A, Bledsoe RD, Cleve P et al. Reassessing the methods of medical record review studies in emergency medicine research. Ann Emerg Med 2005;45:448–51. 10.1016/j.annemergmed.2004.11.021. 15795729

[27] Gilbert EH, Lowenstein SR, Koziol-McLain J et al. Chart reviews In emergency medicine research: Where are the methods? Ann Emerg Med 1996;27:305–8. 10.1016/S0196-0644(96)70264-0. 8599488

[28] Birken SA, Rohweder CL, Powell BJ et al. T-CaST: An implementation theory comparison and selection tool. Implement Sci 2018;13:143. 10.1186/s13012-018-0836-4. 30466450 PMC6251099

[29] R Core Team . *R: A Language and Environment for Statistical Computing*. Vienna: R Foundation for Statistical Computing, 2022.

[30] Cohen J . A coefficient of agreement for nominal scales. Educ Psychol Meas 1960;20:37–46. 10.1177/001316446002000104.

[31] Landis JR, Koch GG. The measurement of observer agreement for categorical data. Biometrics 1977;33:159–74. 10.2307/2529310. 843571

[32] van der Klei VMGTH, Drewes YM, van Raaij BFM et al. Older people’s goals of care in relation to frailty status—The COOP-study. Age Ageing 2024;53:afae097. 10.1093/ageing/afae097. 38796317 PMC11127771

[33] van der Klei VMGTH, van den Bos F, Mooijaart SP et al. A qualitative study regarding older people’s goals of care in relation to frailty status: Finding meaning in ‘smaller things’ in life. Age Ageing 2025;54:afaf022. 10.1093/ageing/afaf022. 39976284 PMC11840562

[34] Chera T, Tinetti M, Travers J et al. “What matters” in the emergency department. Med Care 2024;62:S50–6. 10.1097/MLR.0000000000002053. 39514495 PMC11548826

[35] Ellis HL, Dunnell L, Eyres R et al. What can we learn from 68 000 clinical frailty scale scores? Evaluating the utility of frailty assessment in emergency departments. Age Ageing 2025;54:afaf093. 10.1093/ageing/afaf093. 40253684 PMC12009543

