# Supplementary material for: Assessing what matters most in older emergency department patients
Source: Age Ageing. 2025 Nov 18;54(11):afaf334. doi: 10.1093/ageing/afaf334 (PMC12624858; doi:10.1093/ageing/afaf334)
Supplement: Supplementary_materials_afaf334 [file supplementary_materials_afaf334.docx]

**Assessing What Matters Most in Older Emergency Department Patients**

Appendix Table 1: Questionnaire assessing usability, testability, applicability, and familiarity of the Lim et al. framework. Responses were collected using a four-point rating scale, ranging from 1 (strongly disagree) to 4 (strongly agree).

| Usability |
| --- |
| The framework was easy to use. |
| The structure of the framework is clear. |
| The domains were clearly distinguishable from each other. |
| The categorization of the statements was possible without great effort. |

| Testability |
| --- |
| I had clear criteria for categorizing the statements. |
| The categorization seemed consistent to me. |
| I think that other people would come to similar conclusions. |
| I can give good reasons for my decisions. |

| Applicability |
| --- |
| I think that the framework can be used well in practice. |
| The domains reflect the relevant aspects from patient testimonials. |
| The framework helps to reflect the practice well. |
| The framework is flexible enough for different patient statements. |

| Familiarity |
| --- |
| I was able to understand the logic of the framework straight away. |
| The structure of the framework was easy and quick to learn. |
| I was able to quickly recognize suitable categories for statements. |
| I think that others will be able to learn how to use the framework quickly and confidently. |

Appendix Figure 1: Grouped Column Chart showing the assessment of “What Matters Most” across different age groups. Values are presented as percentages.


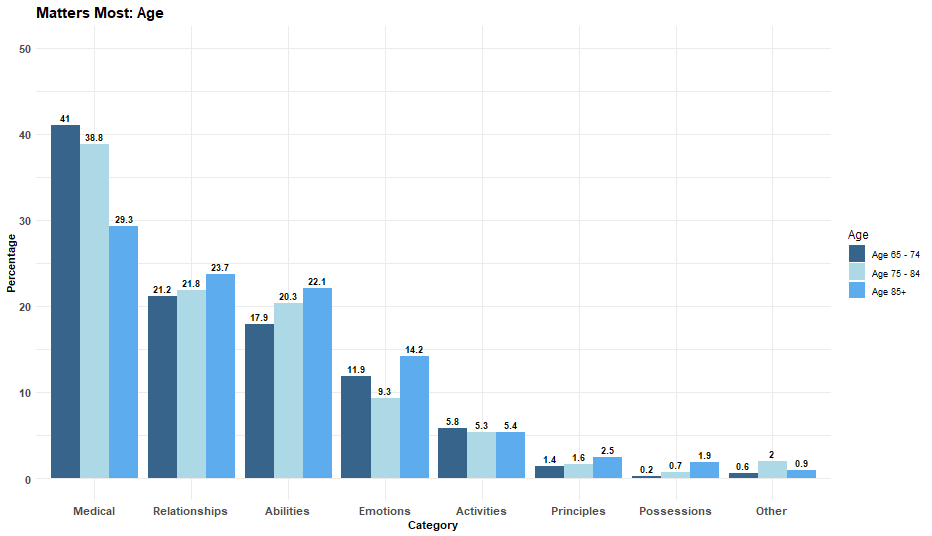


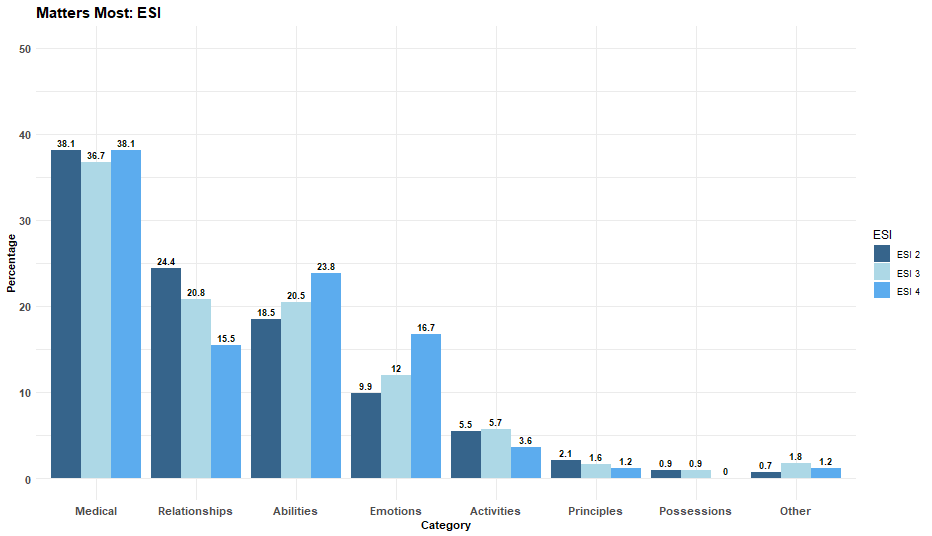
Appendix Figure 2: Grouped Column Chart showing the assessment of “What Matters Most” across different triage levels. Values are presented as percentages.


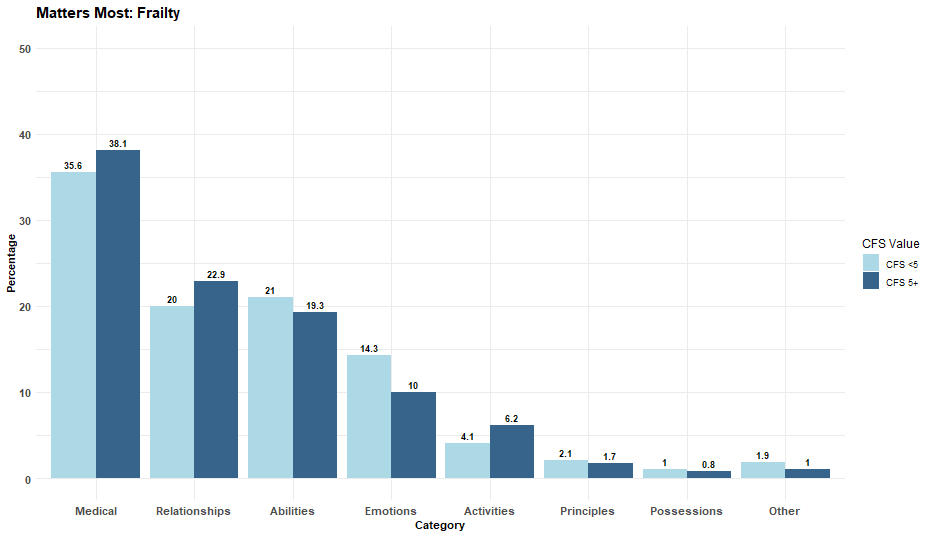
Appendix Figure 3: Grouped Column Chart showing the assessment of “What Matters Most” by frailty status. Values are presented as percentages.

Appendix Figure 4: Network Chart for mean values reflecting 4 domains of the questionnaire. Mean values above 3 were interpreted as indicating general agreement, values around 2 as neutral to slightly negative, and values below 2 as clear disagreement, with 4 being the highest possible score on the scale.


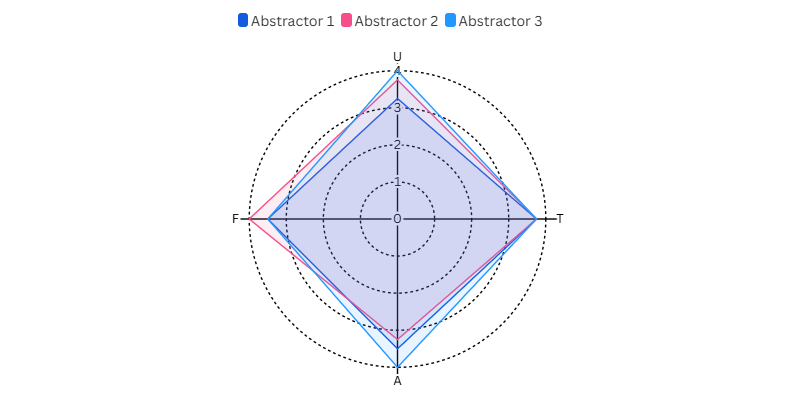


U: Usability, F: Familiarity, T: Testability, A: Applicability
